# Methamphetamine exposure drives cell cycle exit and aberrant differentiation in rat hippocampal-derived neurospheres

**DOI:** 10.3389/fphar.2023.1242109

**Published:** 2023-09-19

**Authors:** Shaomin Wang, Liang Wang, Qian Bu, Qian Wei, Linhong Jiang, Yanping Dai, Ni Zhang, Weihong Kuang, Yinglan Zhao, Xiaobo Cen

**Affiliations:** ^1^ Mental Health Center and National Chengdu Center for Safety Evaluation of Drugs, State Key Laboratory of Biotherapy, Collaborative Innovation Center for Biotherapy, West China Hospital, Sichuan University, Chengdu, China; ^2^ Cell and Immunology Laboratory, Chengdu West China Frontier Pharmatech Co., Ltd., Chengdu, China; ^3^ Mental Health Center, West China Hospital, Sichuan University, Chengdu, Sichuan, China

**Keywords:** methamphetamine, neurospheres, neurodevelopmental toxicity, cell cycle exit, aberrant differentiation

## Abstract

**Introduction:** Methamphetamine (METH) abuse by pregnant drug addicts causes toxic effects on fetal neurodevelopment; however, the mechanism underlying such effect of METH is poorly understood.

**Methods:** In the present study, we applied three-dimensional (3D) neurospheres derived from the embryonic rat hippocampal tissue to investigate the effect of METH on neurodevelopment. Through the combination of whole genome transcriptional analyses, the involved cell signalings were identified and investigated.

**Results:** We found that METH treatment for 24 h significantly and concentration-dependently reduced the size of neurospheres. Analyses of genome-wide transcriptomic profiles found that those down-regulated differentially expressed genes (DEGs) upon METH exposure were remarkably enriched in the cell cycle progression. By measuring the cell cycle and the expression of cell cycle-related checkpoint proteins, we found that METH exposure significantly elevated the percentage of G0/G1 phase and decreased the levels of the proteins involved in the G1/S transition, indicating G0/G1 cell cycle arrest. Furthermore, during the early neurodevelopment stage of neurospheres, METH caused aberrant cell differentiation both in the neurons and astrocytes, and attenuated migration ability of neurospheres accompanied by increased oxidative stress and apoptosis.

**Conclusion:** Our findings reveal that METH induces an aberrant cell cycle arrest and neuronal differentiation, impairing the coordination of migration and differentiation of neurospheres.

## 1 Introduction

Methamphetamine (METH), an amphetamine-type psychostimulant abused worldwide, causes a serious public health issue (Chacho et al., 2019). Long-term METH abuse is frequently linked to severe neuropsychiatric consequences, including anxiety, agitation, hallucinations, paranoia, and psychosis (Jayanthi et al., 2021; Li et al., 2021). METH can cross the placenta barrier to reach the fetus and can be released into breast milk during breastfeeding (Zoubkova et al., 2019). When women of childbearing age use METH, there is a greater chance that they will use it during pregnancy, resulting in maternal and fetal deficits (Slamberova, 2019). Even short-term prenatal METH use is associated with fetal growth restriction, low birth weight, and brain malformations in the newborn (Sankaran et al., 2022). Neuroimaging studies showed that METH-exposed children show smaller subcortical brain volumes and disordered cellular metabolism in the basal ganglia, which is accompanied by neurocognitive deficits (Smith et al., 2001; Chang et al., 2004). Furthermore, METH-exposed children exhibit structural changes in the brain, including reduced volume in the striatum and hippocampus, and altered frontal white matter, suggesting abnormalities in the neuronal and glial development (Won et al., 2002; Li et al., 2021).

Experimental studies show that the administration of METH to pregnant rats causes a markedly increased risk of abortion and maternal death (Matějovská et al., 2014). Prenatal or neonatal METH exposure leads to behavioral hyperactivity or long-term impairments in spatial learning and memory in adolescent mice (Siegel et al., 2011; Ochozková et al., 2019). Poor cognitive performance is also discovered in the adult offspring rats that were exposed to METH during pregnancy (Aghazadeh et al., 2022). These impairments are associated with alterations in hippocampal neurogenesis, synaptic functioning of granule cell neurons (GCNs), or expression of plasticity-related proteins in the dentate gyrus (Takashima and Mandyam, 2018). Growing evidence has discovered that METH exposure negatively impacts the neurogenesis of hippocampal neural stem cells in rodents (Takashima and Mandyam, 2018). At the cellular level, METH-caused neurotoxicity and degenerative effects correlate significantly with specific mitochondrial damage, including damaged mitochondrial morphology, decreased mitochondrial membrane potential, and accumulated oxidative stress (Wong et al., 2008; Potula et al., 2010; Lenzi et al., 2022). In addition to the oxidative stress-induced damage by acting on mitochondria, METH-caused toxicity also involves cell cycle dysregulation in diverse cells, including endothelial cells (Fisher et al., 2015), lymphocyte T (Potula et al., 2018), and DG astrocytes (Jackson et al., 2014). By reducing the levels of progenitors in the S phase of the cell cycle and the number of actively dividing preneuronal neuroblasts while increasing the number of dividing preneuronal progenitor cells, METH exposure eventually inhibits the proliferation, differentiation, maturation, and survival of neural stem cells (Yuan et al., 2011; Takashima and Mandyam, 2018). These results connect the dysregulation of neurogenesis to human brain malformation and dysfunction (Mandyam et al., 2008). In spite of these advances, the molecular mechanisms underlying METH-induced neurodevelopmental deficits have yet to be elucidated.

Current mechanistic studies of METH-induced poor cognitive performance and developmental neurotoxicity *in vitro* mainly employ the two-dimensional (2D) homogenous neural cultures (Dang et al., 2021; Li et al., 2021; Čechová and Šlamberová, 2021; Shrestha et al., 2022). It is quite difficult to mimic the biology of the brain and in particular the dynamic interactions or crosstalk between the numerous cell types and regions in the central nervous system (CNS) using the 2D neural cultural systems. The proliferation of neural stem and progenitor cells (NSCs/NPCs) in liquid culture is able to form floating cell clusters, termed neurospheres (Lobo et al., 2003). They are actively undergoing self-renewal, migration, and early differentiation to form the cytoarchitecture of the brain. In general, cell cycle exit, migration, and differentiation appear to be regulated in a coordinated manner (Takahashi et al., 2007). Progenitor cells in the CNS need to exit the cell cycle and turn on particular processes of differentiation and migration in order to generate new neurons (Nguyen et al., 2006). Hence, neurospheres are increasingly applied to study the mechanisms of different neurodegenerative diseases, infectious diseases, and toxic effect induced by drugs and chemicals (Choi et al., 2013; Prem et al., 2020).

Here, we applied neurospheres derived from embryonic rats to investigate the underlying mechanisms of METH-induced neurodevelopmental toxicity *in vitro.* We found that the disturbance of cell cycle-related pathways contributed to METH-induced small size of neurospheres. The aberrant cell cycle arrest further impaired the coordination of migration and differentiation processes, presenting abnormal differentiation to both neurons and astrocytes and attenuated migration of neurospheres.

## 2 Materials and methods

### 2.1 Chemical and reagents

METH powder (purity of 99.1%) was obtained from the National Institute for the Control of Pharmaceutical and Biological Products (Beijing, China) and formulated into a 10 mM stock solution, stored at 2–8 °C, and diluted into 1.5 mM and 3 mM solutions for neurospheres treatment.

### 2.2 Animals

Sprague-Dawley rats were provided by the Experiment Animal Center of the Sichuan Academy of Chinese Medical Sciences. The rats were housed in an animal room under a standard condition with a 12 h light/dark cycle (light 7:00–19:00) at room temperature (20–25 °C) and relative humidity (40–70%). All experimental procedures and use of the animals were conducted in accordance with the guidelines established by the Association for Assessment and Accreditation of Laboratory Animal Care and the Institutional Animal Care and Use Committee of Sichuan University.

### 2.3 Primary neural stem and progenitor cells (NSCs/NPCs) culture

Neural stem/progenitor cell culture from the embryonic hippocampus of Sprague-Dawley rats was performed according to the procedure from StemCell Technologies with some modifications. Briefly, the hippocampus was dissected from E18 embryos and placed in cold PBS containing 2% glucose on ice. After completing precipitation, the supernatant was discarded and the hippocampal tissue was then resuspended with complete NeuroCult proliferation medium (NeuroCult™ Basal Medium supplemented with 10% NeuroCult™ Proliferation Supplement, 20 ng/mL EGF, 10 ng/mL bFGF, and 2 μg/mL Heparin). The dissected embryonic hippocampus was then mechanically dissociated into individual cells using the glass pipette with a fire-polished tip. After gentle titration 15–20 times, the single-cell suspension was filtered with a 40 μm cell strainer to remove the large cell debris. The single-cell suspension was centrifuged at 200 *g* for 5 min and cell pellets were resuspended with the complete NeuroCult proliferation medium. Lastly, the cell suspension was seeded on the ultra-low adherent 6-well plate with a cell density of 1 × 10^5^ cells per well and incubated in a humidified incubator with 5% CO_2_ at 37°C.

### 2.4 METH treatment

Floating neurospheres were formed and pelleted by centrifuging at 200 g for 5 min after 2 days of culturing. Previous observations suggest a time/dose-dependent effect of METH on the brain (Davidson et al., 2022). Due to the timeline of neurosphere proliferation and differentiation, it was challenging to expose METH at low concentrations for a longer period in order to generate toxicity (Lenzi et al., 2022). Furthermore, according to the previous *in vitro* study, acute high doses of METH induced dopaminergic neuronal autophagy and apoptosis by increasing DNA damage-inducible transcript 4 (DDIT4) expression leading to neurotoxicity. However, such neurotoxic effects were not observed at low concentrations of METH (Li et al., 2017). Therefore, to explore the neurotoxicity induced by METH and verify the previously reported neurotoxicity mechanisms in our neurospheres model (Li et al., 2017), neurospheres were treated with a high concentration of METH at 1.5 mM and 3 mM (final concentration). After METH exposure for 24 h, neurospheres were collected for the subsequent immunoblotting, immunofluorescence, and differentiation analysis.

### 2.5 Differentiation of NSC/NPCs

After METH treatment for 24 h, neurospheres were pelleted by centrifuging at 200 g for 5 min and resuspended with the complete NeuroCult differentiation medium (NeuroCult™ Basal Medium supplemented with 10% NeuroCult™ Differentiation Supplement). The single-cell suspension was pelleted by centrifuging at 200 g for 5 min and resuspended with the complete NeuroCult differentiation medium. Cells were then plated on a matrigel-coated coverslip in a 6-well plate with a density of 2 × 10^5^ cells per well and incubated in a humidified incubator with 5% CO_2_ at 37°C. Cells were differentiated for 7 days, with medium change every 2 days.

### 2.6 Transcriptomic analysis

Neurospheres from three biological replicates were collected for whole genome transcriptional analysis after METH treatment for 24 h. Total RNA was extracted using a miRNeasy Mini Kit (No. 160040742; QIAGEN) according to the manufacturer’s instructions, and 1 μg of total RNA was used to prepare RNA-seq transcriptome libraries using a TruSeq RNA Library Prep kit (No. RS-122-2001; Illumina). After quantifying the concentration with a TBS380 mini-fluorometer, the constructed cDNA libraries were sequenced as 2 × 150 bp read length with the Illumina HiSeq 4000 sequencer instrument. The sequencing reads were trimmed and quality-controlled using SeqPrep and Sickle with default parameters. Lastly, HISAT2 software was applied to perform directional alignment between clean reads and reference genomes, respectively (Kim et al., 2015).

### 2.7 Differential expression analysis and functional enrichment

The gene expression was calculated according to the transcripts per million (TPM) method. The abundance of genes was quantified by adopting RSEM (Li and Dewey, 2011). Genes with fold change (FC) > 2 and *Padj* ≤ 0.05 were considered to be significantly differentially expressed genes (DEGs) (Robinson et al., 2010; Wang et al., 2010; Love et al., 2014). The principal component analysis (PCA) and correlation, scatter, and hierarchical clustering heatmap analysis were performed on the Majorbio website (http://www.www.majorbio.com). In addition, gene ontology (GO) and KEGG enrichment analysis were performed to determine which DEGs were significantly enriched in GO terms and signaling pathways compared to the whole-transcriptome background at Bonferroni-corrected *p*-value ≤0.05. GO and KEGG enrichment analysis was carried out by Goatools and KEGG orthology-based annotation system (KOBAS), respectively (Xie et al., 2011). Protein-protein interaction networks were constructed from all DEGs using Cytoscape with 3.8.2 ClueGO/CluePedia plugin (Cox and Mann, 2008).

### 2.8 RNA extraction and real-time quantitative PCR (RT-qPCR)

Neurospheres were harvested for RT-qPCR assay after METH treatment for 24 h. Total RNA was extracted from the neurospheres using the AxyPrepTM Multisource RNA Miniprep kit (Axygen) according to the manufacturer’s instructions. The single-stranded cDNA was reverse transcribed from extracted RNA with a PrimeScript™ RT reagent kit with gDNA Eraser. The qPCR reaction was performed with SYBR Green MasterMix by a QuantStudio Q5 quantitative PCR instrument (ThermoFisher). The expression of each gene was normalized to the expression of *GAPDH* and analyzed by using the ΔΔCT method. Primers used in this study were listed as follows: *Ccna2* F: 5′-GAGGCAGCCA GACATCACTAACAG-3′, *Ccna2* R: 5′-TTCACAGCCAAATGC AGGGTCTC-3’; *Ccnb1* F: 5′-GTG​CCA​GTG​TGC​GAA​CCA​GAG-3′, *Ccnb1* R: 5′- TGG​GCT​TGG​AGA​GGG​AGT​ATC​AAC-3’; *Ccne2* F: 5′-TCA​AGA​CGC​AGT​AGC​CGT TT-3′, *Ccne2* R: 5′- ATC​TGG​GCT​TCT​TGT​GGA​GAG-3’; *Cdca7* F: 5′-GAGCT TCCCCGGCATATTCT-3′, *Cdca7* R: 5′-CCG​TTC​TGG​GTT​CCT​TCT​GG-3’; *Klf4* F: 5′-CTT​GCA​GCA​GTA​ACA​ACC​CG-3′, *Klf4* R: 5′- CGA​TTC​CTG​GTG​GGA​TAG​CG-3’; *Ccnf* F: 5′-AGC​AAA​CGG​AAG​CGA​GA GAACAG-3′, *Ccnf* R: 5′- GTCCAG GCT​CCA​GTC​CAG​AAG​G-3’; *Nurp1* F: 5′-GCC​ACC​AAC​AGC​CCA​CAC​TTC-3′, *Nurp1* R: 5′- TTC​CGA​CCT​CCA​CCG​ACG​AC-3’; *Notch1* F: 5′-TGC​CAG​GAC​CGT​GAC​AAC​TAC-3′, *Notch1* R: 5′- GCTCGCAC AGTCATCCAGATTG-3’; *GAPDH* F: 5′-AAC​ATC​ATC​CCT​GCC​TCT​ACT​G-3′, *GAPDH* R: 5′- AACATCATCCCTGC CTCTACTG-3’; *Hes1* F: 5′-TCC​AAG​CTG​GAG​AAG​GCA​GA-3′, *Hes1* R: 5′-CTCGTTCATG CACTCGCTGA-3’; *Hes5* F: 5′-TCG​CCA​ATC​GCC​TCC​AGA​G-3′, *Hes5* R: 5′-GGTCCCGAC GCATCTTCTCC-3’; *Hey1* F: 5′-GCT​GAA​GTT​GCC​CGT​TAT​CTG​AG-3′, *Hey1* R: 5′-GCT​GGG​ATG​CGT​AGT​TGT​TGA​G-3’; *Actin* F: 5′-TAC​AAC​CTC​CTT​GCA​GCT​CC-3′, *Actin* R: 5′- GGA​TCT​TCA​TGA​GGT​AGT​CAG​TC-3’.

### 2.9 Western blot

Total proteins were extracted by a whole protein extraction kit (No. BC3710; Solarbio) and protein concentration was measured by a BCA assay kit (No. P0010; Beyotime) according to the manufacturer’s instructions. Equal amounts of proteins were resolved on the 12% sodium dodecyl sulfate-polyacrylamide gel electrophoresis (SDS-PAGE) and transferred to polyvinylidene difluoride (PVDF) membranes. After blocking with non-fat milk, the PVDF membrane was sequentially incubated with the primary and secondary antibodies. Primary antibodies and dilutions used in this study were listed in the following: rabbit anti-CDK2 (1:4000; 22060-1-AP; Proteintech), rabbit anti-CDK4 (1:8000; 11026-1-AP; Proteintech), rabbit anti-CDK6 (1:4000; 19117-1-AP; Proteintech), rabbit anti-Cyclin E1 (1:1000; ab33911; Abcam), mouse anti-Cyclin A (1:500; sc-271645; Santa Cruz Biotechnology), rabbit anti-ɑ-Tubulin (1:2000; #2125; Cell Signaling Technology).

### 2.10 Cell cycle assay

Neurospheres were dissociated into single cells with Accutase™ cell detachment solution (No. 07920; StemCell Technologies). After washing three times with PBS, the dissociated single cells were fixed with 70% ice-cold ethanol overnight at 4 °C. Next, the fixed cells were washed three times with PBS and incubated with 50 μg/mL propidium iodide (PI) in PBS at room temperature for 30 min. The proportion of cells was analyzed by Cytomics FC500 flow cytometer (Beckman Coulter Ltd., United States), and data were analyzed using the CXP Analysis software version 2.2.

### 2.11 Immunofluorescence

Neurospheres were permeabilized and blocked with a blocking solution consisting of 5% normal goat serum and 0.5% TritonX-100 in PBS at room temperature for 1 h. Neurospheres were incubated with the primary antibodies diluted in the blocking solution overnight at 4°C and then incubated with the Alexa-conjugated secondary antibodies at room temperature for 1 h. Neurospheres were further stained with 4′,6-diamidino-2-phenylindole (DAPI) for 5 min and subsequently mounted onto glass slides with ProLong™ Gold Antifade Mountant (No. P36934, ThermoFisher). Images were acquired on a laser scanning confocal microscope (Leica SP8 TCS) with built-in LAS X software (Leica Biosystem). Images were analyzed with ImageJ software (version 1.45). Primary antibodies and dilutions used in this study were listed in the following: rabbit anti-SOX2 (1:400; #3579; Cell Signaling Technology), mouse anti-Nestin (1:400; #4760; Cell Signaling Technology), rabbit anti-Ki67 (1:100; ab15580; BioLegend), mouse anti-β3-Tubulin (1:200; #4466; Cell Signaling Technology), mouse anti-GFAP (1:1000; ab7260; Abcam), rabbit anti-GFAP (1:5000; ab7260; Abcam), mouse anti-Tuj1 (1:1000; ab78078; Abcam).

### 2.12 TUNEL assay

The apoptosis was determined by using a terminal dexynucleotidyl transferase (TdT)-mediated dUTP nick end labeling (TUNEL) assay kit (No. 12156792910; Roche, Switzerland) according to the manufacturer’s instructions. Briefly, the reaction mixture of enzyme solution and labeling solution was freshly mixed at a ratio of 1:9 (v/v). Then, neurospheres were incubated with the reaction mixture for 1 h at room temperature in the dark. And all cell nuclei in neurospheres were subsequently stained with DAPI for 5 min at room temperature protecting from the light. After washing three times with PBS, neurospheres were mounted onto glass slides with ProLong™ Gold Antifade Mountant (No. P36934, ThermoFisher). Apoptosis was quantified by determining the percentage of TUNEL positive-stained (TUNEL+) cells to all DAPI-stained cells using ImageJ software (version 1.45).

### 2.13 Detection of cellular reactive oxygen species (ROS)

Cellular superoxide anions were detected and quantified using dihydroethidium (DHE, D1168; Thermo Fisher Scientific) as a previously reported procedure (Wang et al., 2019). Briefly, neurospheres were dissociated into single cells with Accutase™ cell detachment solution (No. 07920; StemCell Technologies). After washing three times with PBS, the dissociated single cells were pellet and resuspended with prewarmed PBS containing 10 μM DHE. After incubating for 15 min in the dark, cells were precipitated and washed three times with PBS. Lastly, an equal number of cells (1 × 10^5^ cells/well) was added to the 96-well plate. The fluorescence intensity was measured using a Varioskan LUX multimode microplate reader (Thermo Fisher Scientific) with excitation and emission at 510 and 600 nm, respectively.

### 2.14 NPCs migration assay

`Matrigel was used as a supportive matrix in the neurosphere culture. After neurosphere formation, neurospheres were plated on the 6-well plate pre-coated with Matrigel and cultured in neurobasal plus medium (No. A3582901, ThermoFisher) without growth factor. Neurospheres were then cultured in a humidified incubator with 5% CO_2_ at 37°C. To track the migration of NPCs out of neurospheres, images were captured by phase contrast microscopy at 2, 4, and 6 h, respectively. The diameter of migration was measured by ImageJ software (version 1.45). The average outgrowth was quantified by subtracting the inner diameter from the outer diameter as previously reported (Ge et al., 2016).

### 2.15 Statistical analysis

Data are presented as the mean ± SEM. Statistical analysis was carried out through one-way analysis of variance (ANOVA) followed by the Tukey–Kramer test using GraphPad Prism 8 software (version 8). *p* < 0.05 was considered statistically significant. The number of neurospheres or biological replicates that were used for the statistical analysis was presented in the corresponding figure legend of the individual figure.

## 3 Results

### 3.1 METH exposure causes a reduction in the size of neurospheres

**FIGURE 1:**
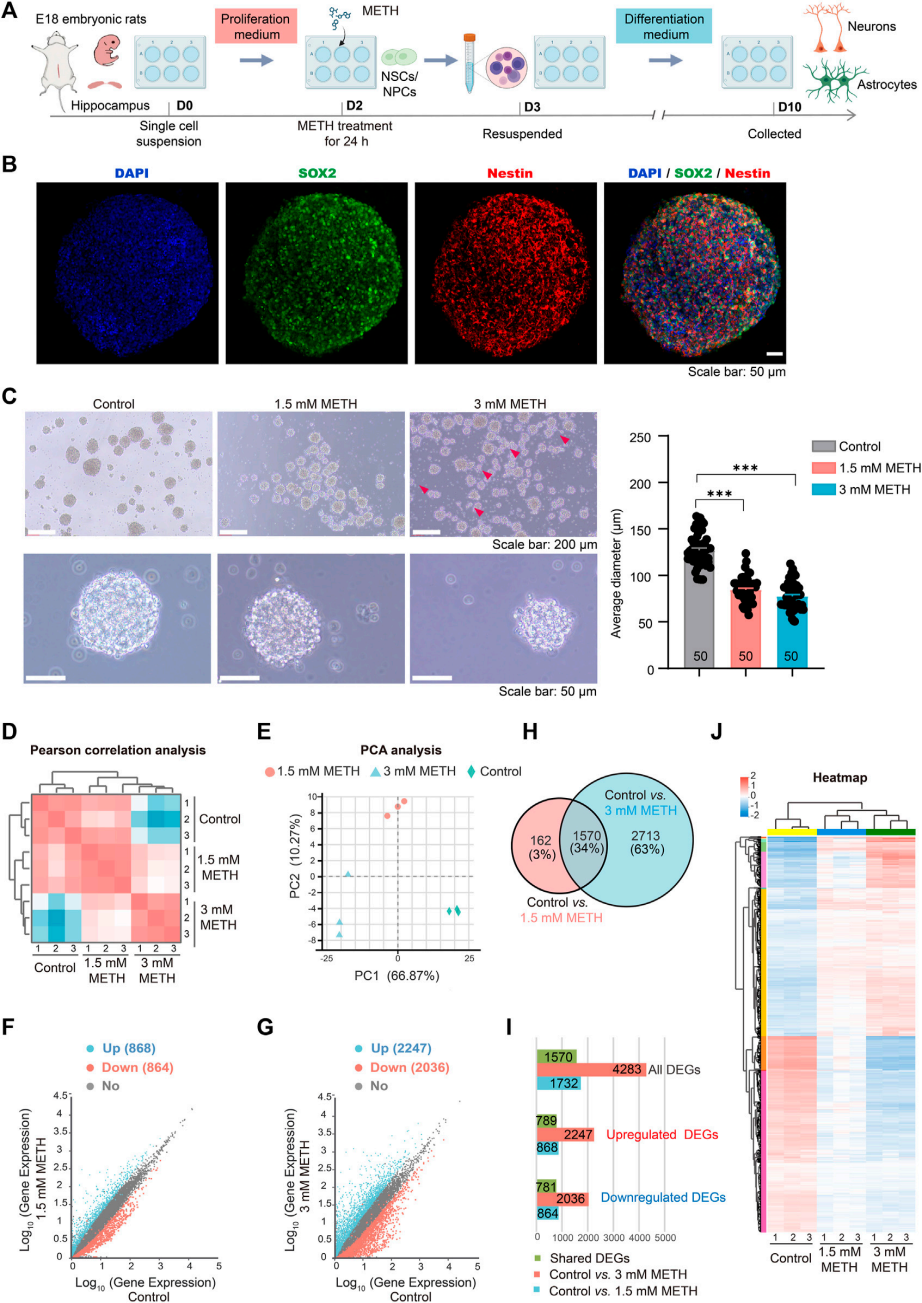
METH treatment reduces the size of neurospheres. **(A)** A schematic diagram of the method for the experimental procedure. **(B)** Neurospheres were immunostained with NSCs/NPCs marker SOX2 and Nestin. Scale bars, 50 µm. **(C)** Phase contrast image of neurospheres treated with METH for 24 h (left panel). Scale bars, 50 µm. The bar graph shows the reduced diameter of neurospheres treated by METH. *n* = 50 per group. Ordinary one-way ANOVA, *F*_treatment_(2,147) = 147.0, *p* < 0.0001. Compared to the control group, ***p < 0.001. **(D)** Pearson correlation analysis of three biological replicates used for differential expression analysis. **(E)** PCA plot shows the differences among the control and METH-treated groups based on changes in gene transcription of neurospheres. **(F)** Scatter plots of all identified genes from RNA-seq analysis after 1.5 mM METH treatment for 24 h. **(G)** Scatter plots of all identified genes from RNA-seq analysis after 3 mM METH treatment for 24 h. For F and G, blue and red dots separately represent significantly upregulated and downregulated genes (Fold change (FC) > 2 and *Padj* ≤ 0.05). **(H)** Venn diagram shows 1570 differentially expressed genes (DEGs) that were identified from both METH-treated groups. **(I)** Bar graph shows the number of DEGs and common DEGs identified from both METH-treated groups. **(J)** The heatmap shows the expression profile of 1570 common DEGs in each biological replicate of samples.

Neural stem and progenitor cells (NSCs/NPCs) were isolated from rat hippocampal tissues of embryonic day 18 (E18), and cultured in the proliferation medium. The floating growing neurospheres appeared after 1–2 days of culturing. On day 2, neurospheres were treated with METH at the final concentration of 1.5 mM and 3 mM, respectively (Li et al., 2017). After METH treatment for 24 h, neurospheres were collected and re-suspended with the differentiation medium without METH, followed by another 7 days for differentiation (Figure 1A). One of the distinguishing features between NSCs and NPCs is the capacity of self-renew and pluripotent differentiation (Hu et al., 2006). To determine these functional properties, SOX2, a well-characterized transcription factor for NSC maintenance and brain development (Zhu et al., 2017; Mercurio et al., 2019), and Nestin, a marker of NSCs/NPCs and radial glial (Onishi et al., 2019), were used to detect the self-renew and pluripotent ability of neurospheres. The immunofluorescent images presented that both SOX2 and Nestin were highly expressed in the neurospheres (Figure 1B), suggesting the high proliferation and differentiation capacity of neurospheres. In contrast to neurospheres in the control group, METH treatment caused a decreased size of neurospheres with increased cell fragments around the neurospheres. The diameter of neurospheres was decreased with the increase in METH concentration (Figure 1C). These results indicate that METH exposure caused the reduced size of the neurosphere.

To explore the molecular mechanisms of reduced neurospheres caused by METH, the whole-genome transcriptional profiles among the control and METH-treated groups were compared using RNA sequencing (RNA-seq). The sample correlation analysis was conducted based on the transcripts per million (TPM) distribution. The Pearson correlation analysis of three biological replicates was almost equal to 1 in each group, indicating the reliability of our RNA-seq results. Furthermore, correlation analysis showed that the differences between the control and METH-treated groups were augmented with the increase in METH concentration (Figure 1D). Similar result was obtained from the unsupervised principal component analysis (PCA) (Figure 1E). Next, the differentially expressed genes (DEGs) between the METH-treated and control groups were separately analyzed. The scatter plots show all the detected genes, with the significantly upregulated and downregulated genes (fold change (FC) > 2 and *Padj* < 0.05) in blue and red, respectively. Compared to the control group, 868 and 2,247 genes were significantly upregulated in the 1.5 mM and 3 mM METH-treated groups, respectively; nevertheless, 864 and 2036 genes were significantly downregulated, respectively (Figures 1F, G; Supplementary Table S1).

Those DEGs that were affected in both METH-treated groups were analyzed. Venn graph showed that 1570 DEGs were dysregulated in both METH-treated groups (Figure 1H; Supplementary Table S1). Among these common DEGs, 781 genes were downregulated and 789 genes were upregulated following METH treatment (Figure 1I; Supplementary Table S1). The expression level of those common DEGs was visualized by heatmap. The results presented a similar expression profile in the METH-treated groups while a distinct difference with the control group. The expression of most DEGs changed gradually in a dose-dependent manner (Figure 1J; Supplementary Table S1). These results suggest that METH can induce neurodevelopmental toxicity dose-dependently.

### 3.2 METH exposure impairs the cell cycle progression of neurospheres

**FIGURE 2:**
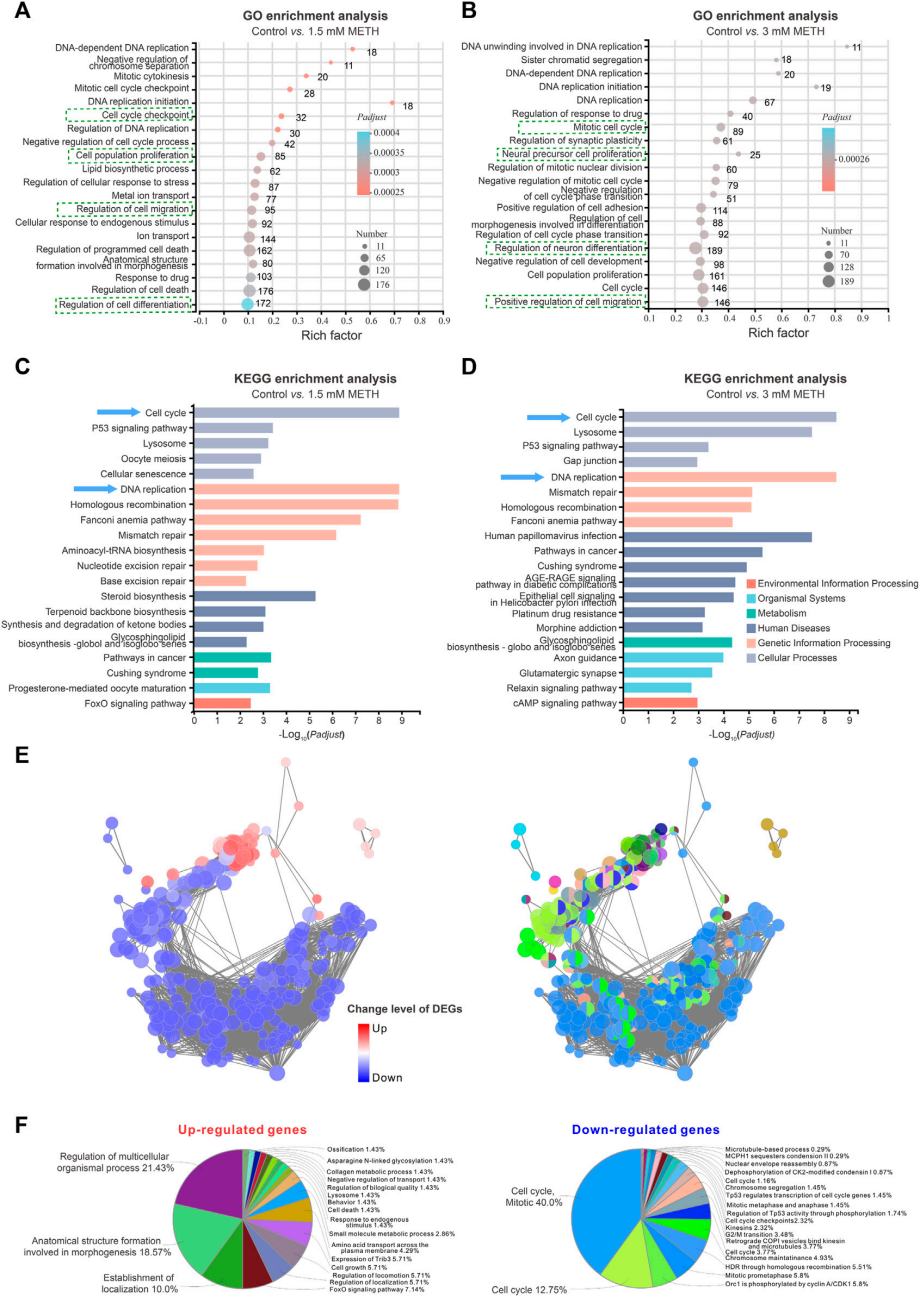
RNA-seq analysis shows an impaired cell cycle progression of neurospheres. **(A)** The bubble graph shows the significantly enriched biological processes in GO enrichment analysis following 1.5 mM METH treatment for 24 h. **(B)** The bubble graph shows the significantly enriched biological processes in GO enrichment analysis following 3 mM METH treatment for 24 h. **(A,B)** the *Padjust* value represents the significance of each term. Red color represents a high degree of enrichment and blue color is the opposite. The size of the bubble represents the number of DEGs. **(C)** The bar graph shows the significantly enriched cell signaling in the KEGG enrichment analysis following 1.5 mM METH treatment for 24 h. **(D)** The bar graph shows the significantly enriched cell signaling in the KEGG enrichment analysis following 3 mM METH treatment for 24 h. **(E)** Network enrichment analysis of DEGs that were identified in both METH-treated groups. For the left panel, the color code of nodes corresponds to the upregulated and downregulated DEGs. For the right panel, the color code of nodes corresponds to the changes in the functional group to which they belong. **(F)** The pie charts show the percentage of each enriched functional group in the network analysis. The enriched functional groups of upregulated and downregulated DEGs are displayed in the left and right panels, respectively.

To investigate the disturbed cell signaling in response to METH, all DEGs were subjected to gene ontology (GO) enrichment and KEGG analysis, respectively. GO enrichment analysis discovered that several biological processes were significantly enriched in both 1.5 and 3 mM METH groups. As shown in Figures 2A, B, several biological processes involved in DNA replication, cell cycle, cell migration, and cell differentiation were found to be affected by METH. KEGG analysis based on these changed biological processes further revealed that several cell signaling were enriched in both METH-treated groups, such as cellular processes (e.g., cell cycle, p53 signaling pathway, lysosome), genetic information processing (e.g., DNA replication, mismatch repair, homologous recombination), and regulating organismal systems (e.g., axon guidance, glutamatergic synapse, GABAergic synapse) (Figures 2C, D). Notably, cell cycle and DNA replication were the two most affected cell signalings that were enriched. In agreement with previous studies, cell cycle-related signaling pathways displayed significantly high variations following the METH treatment (Jackson et al., 2014). These results indicate the reduced size of neurospheres caused METH may be attributed to the disturbed cell cycle.

How those signaling pathways were disturbed by METH was further addressed. The cell signaling involved in DEGs was visualized via ClueGO/CluePedia plugin from Software Cytoscape (version 3.8.2). Although a similar number of upregulated and downregulated DEGs were identified in the METH-treated groups, the downregulated DEGs dominated the enriched signaling pathways (Figure 2E). Furthermore, almost 80% of downregulated DGEs-enriched pathways were involved in the cell cycle. As shown in the pie chart, the top three downregulated DGEs-enriched pathways were cell cycle, mitotic (40%), and cell cycle (12.75%); moreover, Orc1 is phosphorylated by cyclin A/CDK1 (5.8%). In contrast to the downregulated DGEs, the upregulated DGEs-enriched pathways were diverse, and the top three enriched pathways are involved in the regulation of multicellular organismal processes (21.43%), anatomical structure formation (18.57%), and establishment of localization (10.0%) (Figure 2F). Taken together, these findings suggest that METH exposure may cause cell cycle arrest during the proliferation period of NSCs/NPCs.

### 3.3 METH exposure leads neurospheres to exit the cell cycle from the G0/G1 phase by inhibiting the G1/S transition

**FIGURE 3:**
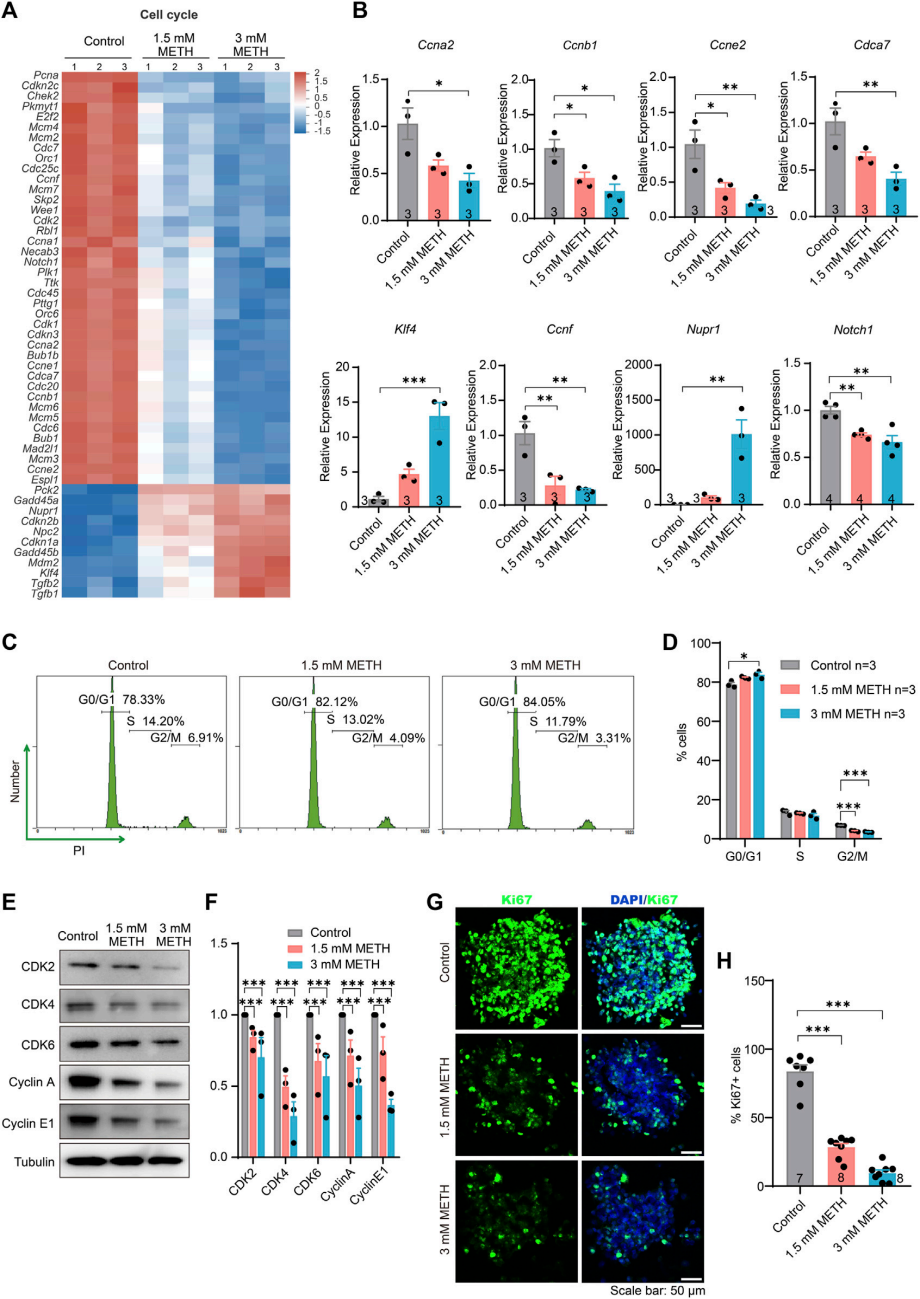
METH exposure causes cell cycle arrest in the G0/G1 phase by inhibiting the G1/S transition. **(A)** The heatmap shows the expression profile of cell cycle-involved DEGs. **(B)** The bar graphs display the transcriptional levels of DEGs using the RT-qPCR assay. *n* = 3 per group. Ordinary one-way ANOVA, for *Ccna2*: *F*_treatment_(2,6) = 7.864*, p =* 0.0211; *Ccnb1*: *F*_treatment_(2,6) = 9.306*, p =* 0.0145; *Ccne2*: *F*_treatment_(2,6) = 11.76*, p* = 0.0084; *Cdca7*: *F*_treatment_(2,6) = 10.39, *p* = 0.0113; *Klf4*: *F*_treatment_(2,6) = 25.53*, p* = 0.0012; *Ccnf*: *F*_treatment_(2,6) = 13.43, *p* = 0.0061; *Nurp1*: *F*_treatment_(2,6) = 21.83, *p = 0.0018*; *Notch1*: *F*_treatment_(2,9) = 13.87, *p =* 0.0018. Compared to the control group, *p < 0.05, **p < 0.01, and ***p < 0.001. **(C)** Representative flow cytometric data shows the distribution of cell cycle using PI staining after METH treatment for 24 h. The percentage of each cell phase is marked in the images. **(D)** The bar graph displays the percentage of each cell phase in the **(C)** *n* = 3 per group, Ordinary one-way ANOVA, for G0/G1 phase: *F*_treatment_(2,6) = 8.327, *p* = 0.0186. Compared to the control group, *p < 0.05. For G2/M pahse: *F*_treatment_(2,6) = 159.8, *p* < 0.0001. Compared to the control group, ***p < 0.001. **(E)** Immunoblotting images show the changed level of various cyclin and cyclin-dependent kinases. **(F)** The bar graph displays the related protein level of cyclin and cyclin-dependent kinases in the **(E)** *n* = 3 biological replicates per group. Two-way repeated measured ANOVA, followed by Dunnett’s multiple comparisons test, *F*_protein_(4,32) = 2.893, *p* = 0.0376; *F*_treatment_(2,6) = 94.05, *p* < 0.0001; *F*_interaction_(6,32) = 0.4104, *p* = 0.8666. Compared to the control group, ***p < 0.001. **(G)** Representative images present the proliferative capacity of neurospheres using Ki67 staining after METH treatment for 24 h. Scale bar, 50 μm. **(H)** The bar graph shows the quantified results of Ki67-positive stained cells to the total cells. *n* = 7–8 per group. Ordinary one-way ANOVA, *F*_treatment_(2,20) = 129.7, *p* < 0.0001. Compared to the control group, ***p < 0.001.

The cell cycle is a series of precisely timed and carefully regulated stages of growth, DNA replication, and division into two genetically identical cells including two major phases interphase and mitosis (Lubischer, 2007). We then look into which stage of the cell cycle was arrested by METH. Firstly, those DEGs implicated in the cell cycle were investigated. The expression profile of cell cycle-involved genes was presented in the heatmap. The majority of those DEGs were actually downregulated upon METH exposure (Figure 3A). Then, the altered level of several DEGs that are critical for cell cycle progression was validated through RT-qPCR analysis (Figure 3B). Compared to the control group, genes of cyclin A2 (*Ccna2*), cyclin B1 (*Ccnb1*), cyclin E2 (*Ccne2*), cyclin F (*Ccnf*), Notch receptor 1 (*Notch1*), and cell division cycle-associated 7 (*Cdca7*) were significantly downregulated by METH, while the expression of KLF transcription factor 4 (*Klf4*) and nuclear protein 1(*Nupr1*), which are important for cell cycle progression (Choi and Roh, 2018; Li et al., 2020), were significantly upregulated. These results indicated that METH caused an aberrant cell cycle progression of neurospheres.

Moreover, the effect of METH on the cell cycle distribution of neurospheres was analyzed by flow cytometry with propidium iodide (PI) staining. The results showed that compared to the control, METH exposure resulted in a reduction in the percentage of cells in the S and G2/M phases but an increase in the G0/G1 phase (Figures 3C, D), indicating that METH exposure caused a cell cycle arrest at G0/G1 phase. We further measured the expression levels of various cyclin proteins and cyclin-dependent kinases that control the progression of cells by regulating the cell cycle. The results showed that the levels of the proteins involved in the G1/S transition were significantly reduced by METH, such as CDK2, Cyclin E1, CDK4/6, and the G2/M transition, Cyclin A (Figures 3E, F). Similar to a previous finding that METH protracts G1/S phase transition in the T cells (Potula et al., 2018), cell cycle arrest caused by METH may also be associated with the impairment of G1/S transition. Lastly, the cell proliferation capacity following METH exposure was detected by immunostaining Ki67, a marker for cell proliferation protein. By counting the number of Ki67-positive (Ki67+) cells and DAPI-stained cells, we found that the percentage of Ki67+ cells was diminished in the METH-treated neurospheres compared with control neurospheres, suggesting an attenuated proliferation capacity of neurospheres (Figures 3G, H). Collectively, these results suggest that METH causes cell cycle arrest by impairing G1/S transition, resulting in lower self-renew and cell division.

### 3.4 METH induces aberrant cell differentiation of neurospheres

**FIGURE 4:**
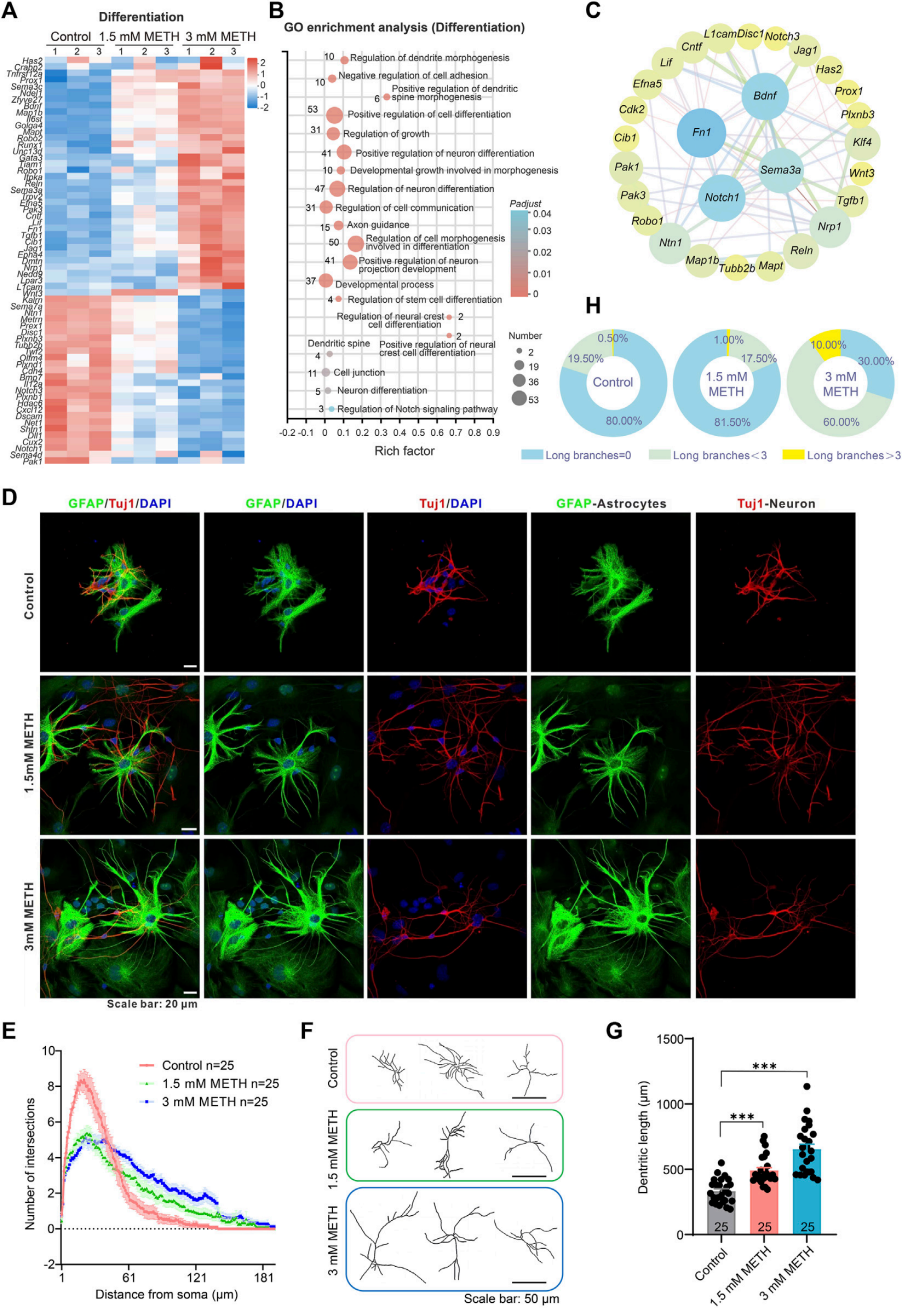
METH treatment causes aberrant neuronal differentiation. **(A)** The heatmap shows the expression profile of cell differentiation-involved DEGs. **(B)** The bubble graph shows the significantly enriched biological processes in GO enrichment analysis using cell differentiation-involved DEGs. The *Padj* value represents the significance of each term. Red color represents a high degree of enrichment and blue color is the opposite. The size of the bubble represents the number of DEGs. **(C)** STRING analysis shows protein-protein interactions of the DEGs from the cell cycle and cell differentiation. **(D)** Representative images show the morphology of GFAP-labeled astrocytes and Tuj1-labeled neurons after differentiation culture. Scale bars, 20 μm. **(E)** The line graph shows changes in dendritic arborization in the Sholl analysis after METH treatment for 24 h *n* = 25 neurons per group. Ordinary one-way ANOVA, *F*_treatment_(2,570) = 3.5, *p* = 0.0309. Compared to the control group, *p < 0.05. **(F)** Representative images show the effect of METH on neuronal complexity. Scale bars, 50 μm. **(G)** The bar graph shows the effect of METH on the total dendritic length. *n* = 25 neurons per group. Ordinary one-way ANOVA, *F*_treatment_(2,72) = 34.58, *p* < 0.0001. Compared to the control group, ***p < 0.001. **(H)** The pie chart shows METH induces changes in astrocyte morphology, *n* = 200 astrocytes per group.

The effect of METH on the cell differentiation of neurospheres was examined. The profile of those DEGs enriched in the GO term was displayed in the heatmap. The change level of the involved genes was presented in a dose-dependent manner (Figure 4A). GO enrichment analysis based on those DEGs showed a number of neural differentiation pathways enriched, such as regulation of dendritic spine morphogenesis, axon guidance, and positive regulation of cell differentiation. Among these GO terms, positive regulation of neural crest cell differentiation, positive regulation of dendritic spine morphogenesis, and regulation of cell morphogenesis involved in differentiation were the top three enriched GO terms (Figure 4B). To explore the relationship of DEGs between cell cycle and cell differentiation, a protein-protein interaction (PPI) analysis was conducted using the STRING tool (https://stringdb.org/) and Cytoscape software (Cytoscape 3.8.2) (Szklarczyk et al., 2017). The interaction network presented that cell cycle-related DEGs, *Notch1, klf4,* and *Cdk2* were co-expressed with cell differentiation-related DEGs, e.g., *Notch3, Runx1, Wnt3* (Figure 4C). The decreased *Notch1 and Notch3* suggest the inhibition of Notch signaling, a pathway playing a central role in various aspects of development and cellular differentiation, for instance, the maintenance of the undifferentiated state and cell fate decision (Yoon and Gaiano, 2005; Harada et al., 2021). Analysis of the downstream genes of Notch signaling, *Hes1*, *Hes5*, and *Hey1* also found reduced expression upon METH treatment (Supplementary Figure S1A). Collectively, these results suggest that METH exposure may accelerate cell cycle exit and then cause abnormal cell differentiation.

To investigate the effect of METH on cell differentiation, the morphology of neurons and astrocytes was visualized after differentiation culturing on day 10. For Tuj1-stained differentiating and mature neurons, the dendrites of METH-treated neurons were obviously longer than those of the control group. Additionally, the complexity of neurons was also altered by METH. Compared to the multiple dendrites from the soma in the control group, METH markedly decreased the dendrites number from the soma but increased the branches from the primary dendrites (Figure 4D). To quantify changes in neuronal morphology, we traced the dendrites of individual-stained neurons and performed Sholl analysis to assess dendritic arborization. The line profile displayed that the dendritic branching complexity was indeed changed by METH, exhibiting reduced branches within 60 μm distance area from the soma while increased branches beyond that area (60–180 μm distance area from the soma) (Figures 4E, F). Similarly, the total dendritic length was also extended by METH (Figure 4G). These results indicate that METH promoted abnormal neuronal differentiation. Except for the aberrant differentiation of neurons, we also noticed the changes in the morphology of GFAP-labeled astrocytes. Normally, the astrocytes in the cultured neurospheres present typical star-shaped morphology with large round somata extending thick and short processes that branch out into several small processes. Nevertheless, METH treatment led to an increase in the astrocytes with more elongated and thin processes (Figure 4D). Then, astrocytes were classified into three groups based on their processes: those with no branches, those with long branches below 3, and those with long branches above 3 (Supplementary Figure S1B). The quantified results showed that the percentage of astrocytes with long processes above 3 increased from 0.5% in the control neurospheres to 10% in the 3 mM METH-treated neurospheres (Figure 4H). According to the subpopulation of astrocytes in the CNS (Matyash and Kettenmann, 2010; Köhler et al., 2021; Köhler et al., 2023), the increased ratio of astrocytes with elongated branches upon METH exposure suggests the possibility of decreased protoplasmic astrocytes while with increased fibrous astrocytes in METH-treated neurospheres. Taken together, METH can cause an abnormal development of neurospheres, which may be attributed to the aberrant cell cycle exit and cell differentiation.

### 3.5 METH induces aberrant neuronal migration in neurospheres

**FIGURE 5:**
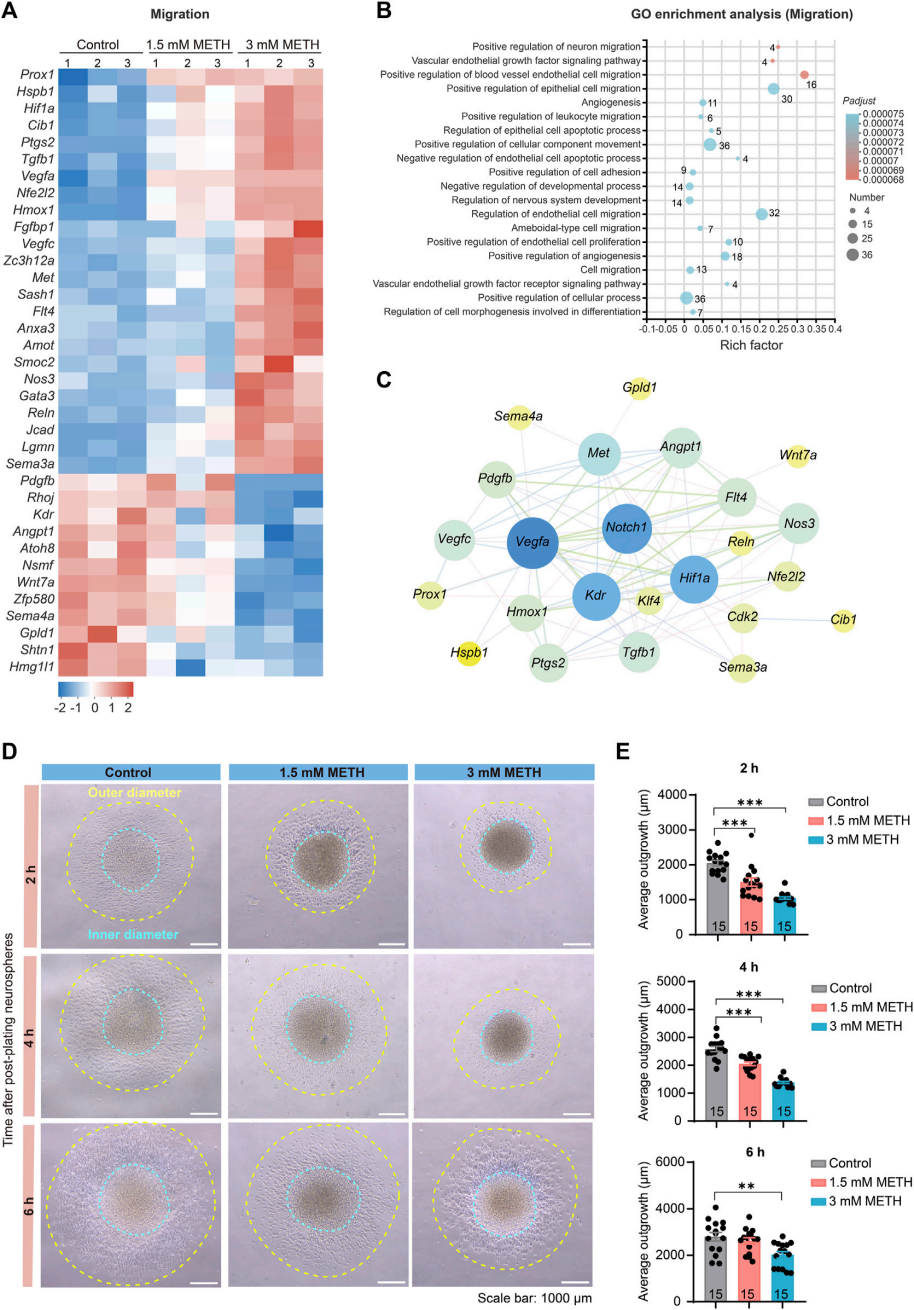
METH treatment causes aberrant neuronal migration. **(A)** The heatmap shows the expression profile of cell migration-involved DEGs. **(B)** The bubble graph shows the significantly enriched biological processes in GO enrichment analysis using cell migration-involved DEGs. The *Padj* value represents the significance of each term. Red color represents a high degree of enrichment and blue color is the opposite. The size of the bubble represents the number of DEGs. **(C)** STRING analysis shows protein-protein interactions of the DEGs from the cell cycle and cell migration. **(D)** Phase contrast images show the migration distance of cells from neurospheres after being plated on the Matrigel pre-coated 6-well plates and visualized at 2, 4, and 6 h, respectively. Scale bars, 1000 μm. **(E)** The bar graph shows the effect of METH on the cell migration distance at the indicated time. *n* = 15 neurospheres per group. Ordinary one-way ANOVA, for 2 h, *F*_treatment_(2,42) = 34.94*, p* < 0.0001; 4 h, *F*_treatment_(2,42) = 74.26, *p* < 0.0001; 6 h, *F*_treatment_(2,42) = 6.833, *p* = 0.0027. Compared to the control group, **p < 0.01 and ***p < 0.001.

NSCs/NPCs migrate to the site of brain development or injury, where they undergo differentiation into specific cell types, such as neuron and glial cells (Csobonyeiova et al., 2019; Akter et al., 2021). To investigate whether the abnormal astrocyte morphology resulted from the altered cell migration in neurospheres, we analyzed those DEGs enriched in the GO term, called regulation of cell migration. The heatmap presented that most of those DEGs involved in cell migration underwent extensive changes following METH treatment (Figure 5A). GO analysis showed a number of migration-related pathways enriched, such as neuron migration, cell adhesion, and cellular component movement (Figure 5B). Moreover, the relationship of DEGs between cell cycle and cell migration was explored using the PPI analysis. The results showed that cell cycle-related DEGs, *Notch1*, *Klf4*, and *Cdk2* formed an interaction network with cell migration-related DEGs, such as *Wnt7a*, *Pdgfb*, *Vegfa* (Figure 5C), further demonstrating that METH-dysregulated cell cycle exit was accompanied with aberrant cell migration.

To verify whether METH exposure resulted in changes in cell migration of neurospheres, we applied an adherent culture system to investigate the effect of METH on cell migration capacity. After plating neurospheres on Matrigel-coated plates, the distance of cells migrated out of neurospheres was traced at 2 h, 4 h, and 6 h post-plating, respectively. The phase contrast images presented the diminished cell migration distance in the METH group (Figure 5D). We then separately measured the inner diameter and outer diameter, and the average outgrowth was obtained by subtracting the inner diameter from the outer diameter. The decreased distance of outgrowth indicated that METH attenuated the migration ability of NSCs/NPCs out of neurospheres (Figure 5E). Taken together, METH exposure attenuated the migration of NSCs/NPCs to the target site of brain development, which may attribute to the abnormal cell differentiation.

### 3.6 METH exposure eventually causes enhanced oxidative stress and apoptotic cell death in neurospheres

**FIGURE 6:**
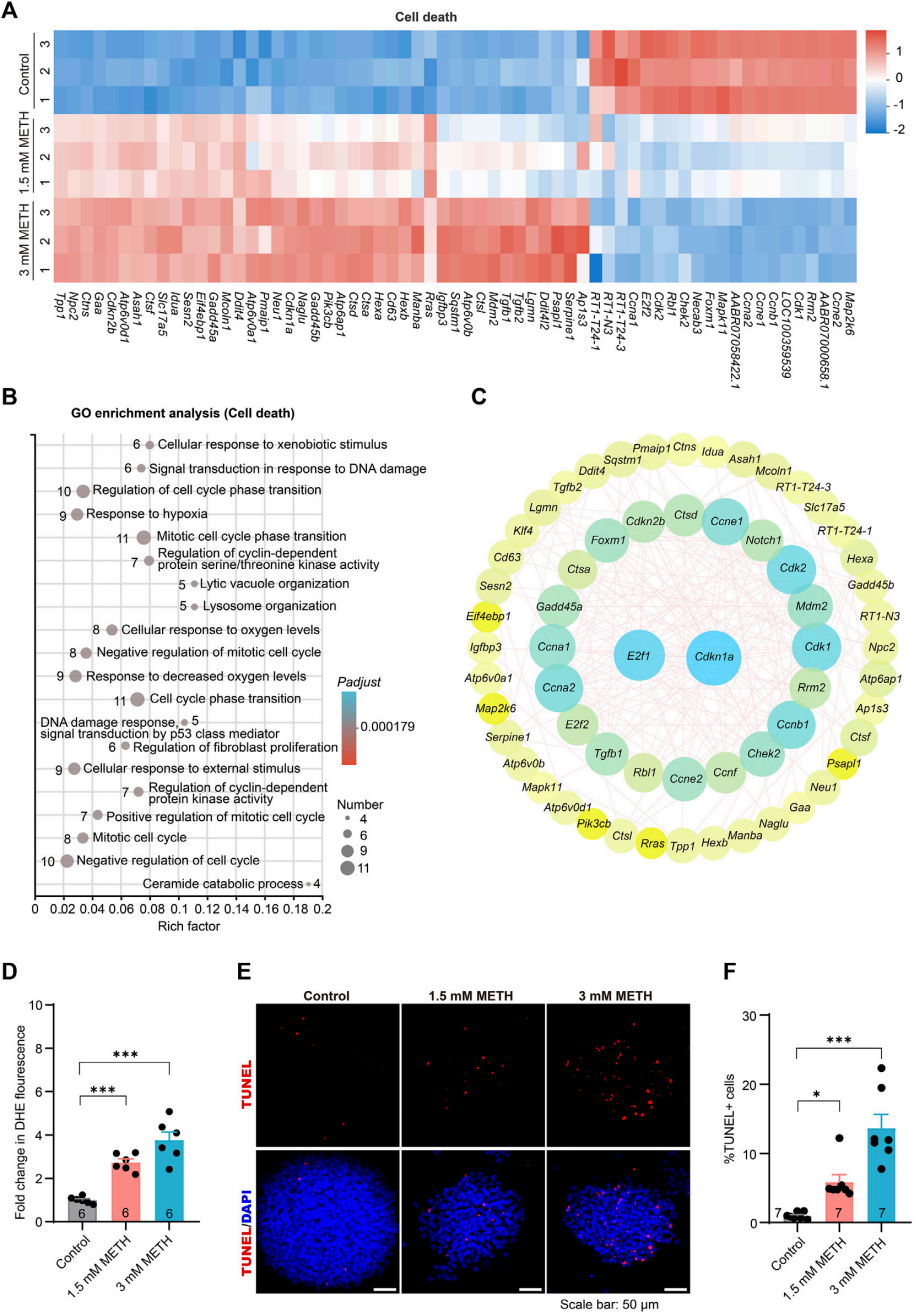
METH treatment causes enhanced oxidative stress and apoptotic cell death. **(A)** The heatmap shows the expression profile of cell death-involved DEGs. **(B)** The bubble graph shows the significantly enriched biological processes in GO enrichment analysis using cell death-involved DEGs. The *Padj* value represents the significance of each term. Red color represents a high degree of enrichment and blue color is the opposite. The size of the bubble represents the number of DEGs. **(C)** STRING analysis shows protein-protein interactions of the DEGs from cell death. **(D)** The bar graph display fold changes in DHE fluorescence intensity. *n* = 6 per group. Ordinary one-way ANOVA, *F*_treatment_(2,15) = 32.36, *p* < 0.0001. Compared to the control group, ***p < 0.001. **(E)** Representative images present the apoptosis of neurospheres using TUNEL staining after METH treatment for 24 h. Scale bar, 50 μm. **(F)** The bar graph shows the quantified results of TUNEL-positive stained cells to the total DAPI-stained cells. *n* = 7 per group. Ordinary one-way ANOVA, *F*_treatment_(2,18) = 24.14, *p* < 0.0001. Compared to the control group, *p < 0.05 and ***p < 0.001.

Previous study has found that high doses of METH treatment caused dopaminergic neuronal autophagy and apoptosis (Li et al., 2017). In this study, neurospheres were also subjected to high doses of METH for 24 h. Hence, to systematically assess whether the reported neurotoxicity occurred in the neurospheres, those DEGs involved in cell death, including the P53 signaling pathway, lysosome, and cellular senescence, were further analyzed. The heatmap presented that most of those DEGs involved in cell death were upregulated in a dose-dependent manner following METH treatment (Figure 6A), indicating the progress of cell death. GO enrichment analysis using those DEGs enriched a number of GO terms involving oxidative stress and DNA damage. The enrichment of those GO terms, such as response to hypoxia, lysosome organization, and cellular response to oxygen level, were supposed to be associated with apoptotic cell death (Figure 6B). Moreover, the PPI analysis found the interaction of diverse cell cycle involved DEGs (such as *Ccna2*, *Cdkn1a*, *Ccnf*, and *Ccne2*) with the *Ddit4* (Figure 6C), a gene involved in hypoxia-triggered apoptosis that is significant upregulated in METH-induced neurotoxicity (Li et al., 2017). The occurrence of the previously reported METH-induced neurotoxicity in neurospheres supports the validity of the toxic mechanism of cell cycle exit and abnormal differentiation.

Lastly, to determine whether METH exposure led to oxidative stress and apoptosis in neurospheres, the cellular oxidative stress and cell death of neurospheres were separately evaluated. Analysis of cellular superoxide anions using a fluorescent probe DHE found that METH exposure resulted in increased cellular oxidative stress (Figure 6D). The apoptotic cell death was determined using the TUNEL assay, a commonly used method to detect apoptosis *in situ*. In contrast to a very low number of TUNEL-positive (TUNEL+) cells in the control neurospheres, the percentage of TUNEL+ cells was evidently increased in a dose-dependent manner upon METH treatment (Figures 6E, F). Collectively, these results indicate that METH causes cell cycle arrest, abnormal cell differentiation, and attenuated cell migration eventually accompanied by increased oxidative stress and apoptotic cell death.

## 4 Discussion

Prenatal METH exposure has been linked to a wide range of CNS toxicity in the offspring, including impaired executive functioning, poor inhibitory regulation of behavior, defects in gross motor development, and growth restriction in fetuses. Brain imaging reveals structural abnormalities in prenatally METH-exposed children (Chang et al., 2004; Derauf et al., 2009). In humans, low doses of METH can improve mood and cognitive functions by acutely increasing extracellular dopamine, while high doses of METH produce severe cellular toxicity and behavioral reinforcing effects by increasing mesolimbic dopamine release (Cruickshank and Dyer, 2009; Moszczynska and Callan, 2017). Moreover, aberrant cerebral hemodynamics as well as neurotransmitter release and uptake are also thought to be involved in METH-caused neurodevelopmental toxicity (Dixon, 1989; Sankaran et al., 2022). At the cellular level, METH-caused neurotoxicity and degenerative effects correlate with oxidative stress/mitochondrial damage (Wong et al., 2008; Potula et al., 2010; Lenzi et al., 2022) and cell cycle dysregulation (Jackson et al., 2014; Fisher et al., 2015; Potula et al., 2018). In this study, through 3D cultured rat neurospheres, we also found that METH exposure caused dysregulation of the cell cycle in neurospheres. More importantly, we revealed that the cell cycle exit during the early neurodevelopment stage of neurospheres might promote aberrant cell differentiation and attenuated cell migration ability accompanied by increased oxidative stress and apoptosis. Collectively, these findings add further information about the underlying mechanism of METH-caused neurotoxicity.

Mammalian behavior is thought to emerge from complex and dynamic cellular interactions among a cast of specialized cells in the CNS that are able to receive, process, and send information (Marton and Pasca, 2020). Neurospheres are 3D structures that are generated *in vitro* from the division and differentiation of NSCs/NPCs, which can better mimic the initial organizational environment, including tissue-specific structure, mechanical and biological characteristics, intercellular communication and signal transmission, and differentiation capabilities (Kobolak et al., 2020). Therefore, the neurosphere is a valuable tool to study brain development and develop new treatments for neurological disorders *in vitro* (Jensen and Parmar, 2006). In this study, neurospheres derived from embryonic rats were used to investigate the underlying mechanisms of METH-induced neurodevelopmental toxicity. Due to the timeline of neurosphere proliferation and differentiation, it was challenging to expose METH at low concentrations for a longer period in order to generate toxicity (Lenzi et al., 2022). Hence, to ensure the toxic effects caused by METH, neurospheres were treated with high concentrations of METH for a short period. As previously reported METH-induced neurotoxicity in PC12 and SH-SY5Y cells, the expression level of DDIT4, a gene involved in hypoxia-triggered apoptosis, is profoundly upregulated after treating PC12 and SH-SY5Y cells with 3 mM and 2 mM METH for 24 h, respectively. However, DDIT4-related effects were not observed after 1 μM METH treatment (Li et al., 2017). In this study, we referred to the reported toxic effects caused by METH at 24 h and thus set the concentration of METH at 1.5 mM and 3 mM METH. Similar to the previously discovered upregulated level of DDIT4, after treating the neurospheres with 1.5 mM and 3 mM METH for 24 h, the expression level of *Ddit4* was also elevated, demonstrating the success of METH-induced neurotoxicity in neurospheres. Except for the upregulated *Ddit4* level, the oxidative stress and apoptotic death were also evident with the increase in METH concentration. Moreover, we additionally found that METH markedly disturbed the expression of the genes associated with cell cycle progression, which resulted in cell cycle arrest and eventually led to abnormalities in both differentiation and migration of neurospheres. Although the screened DEGs in this study were differently changed compared to the reported DEGs in METH-treated hippocampus *in vivo* (Zoubková et al., 2019; Li et al., 2022), the identified DEGs in neurospheres were not only validated by the qRT-PCR but the DEGs-enriched cell signaling was also investigated, demonstrating the critical role of altered genes in METH-caused toxicity. Hence, our findings shed light on the mechanisms underlying METH-induced neurodevelopmental deficits.

In the present study, METH exposure significantly reduced the size of neurospheres, which was supported by the reduced expression level of Ki67, a proliferative capacity biomarker of hippocampal NSCs/NPCs (Ekthuwapranee et al., 2015). A similar result was reported in multiple patients’ iPSC-derived neurospheres as well as Zika virus-infected neurospheres (Brennand et al., 2015; Garcez et al., 2016; Lee et al., 2020). The common characteristic of reduced neurospheres is linked to abnormalities in brain function, ranging from neurodevelopment delay, to neurological dysfunction, and mental diseases. Through enriching the DEGs involved in GO term, a large number of downregulated genes were enriched in the cell cycle, such as cell cycle activity and DNA replication (e.g., cell cycle checkpoint, DNA replication initiation). Analysis of cell cycle distribution and the altered expression of checkpoint proteins further indicated that METH-caused cell cycle arrest was mainly halted to the G0/G1 phase. Interestingly, METH-caused disruption of the cell cycle is presented not only in the CNS but also in the blood-brain barrier endothelial cells, astrocytes, and T cells (Jackson et al., 2014; Fisher et al., 2015; Potula et al., 2018).

NSCs/NPCs are kinds of self-renewing cells that are able to differentiate into various cell types in the CNS, including neurons, astrocytes, and oligodendrocytes. Notch and the downstream genes Hes1 and Hes5 were reduced by METH, indicating that METH exposure decreased cell proliferation while promoting cell differentiation. Further evidence for altered cell differentiation or an imbalance in cell fate decisions comes from the reduced Hey1, a target gene regulated by Notch signaling and essential for cell fate decisions (Yoon and Gaiano, 2005; Harada et al., 2021). Pluripotent stem cells in G1 are prone to differentiation, while pathways operating in S and G2 phases may actively repress the dissolution of the pluripotent state (Gonzales et al., 2015). The pluripotency of pluripotent stem cells is mainly enforced by cell cycle proteins. Hence, the loss or the mutation of cell cycle proteins, such as CDK2 and cyclin E, results in the loss of the pluripotent state and triggered differentiation (Coronado et al., 2013; Liu et al., 2019). We also observed that METH caused decreased expression of both cyclin and CDK checkpoint proteins, suggesting a precocious development resulting from abnormal cell cycle exit and the initiation of cell differentiation. Furthermore, METH significantly altered the morphology of neuronal cells of neurospheres, which presented a decreased number of primary branches from the soma but increased secondary branches. Additionally, the total length of dendrites was also extended by METH treatment. Considering that neuronal morphology is a fundamental feature of neural connection which is important to balance dendritic growth and pruning to maintain proper synaptic connectivity in the brain, we speculate that the excessive dendritic growth may link METH exposure to diverse neurological disorders, such as autism spectrum disorder (ASD), Alzheimer’s disease (AD), and schizophrenia (Coleman and Flood, 1987; Moyer et al., 2015; Weir et al., 2018). For example, an overcompensation of dendritic growth was observed in the early stages of AD, which results in the formation of aberrant synaptic connections and thus leads to cognitive impairment (Tang et al., 2023).

We found that METH exposure caused aberrant differentiation of neurons in the neurospheres. As neuronal differentiation can be triggered by the local environment, such as the mechano-sensing process and cell-substrate interaction during the cell migration (Gu et al., 2009), we speculate that local environments might participate in this process during cell migration. Studies are needed to address this point in the future. In addition, previous studies have reported that METH-caused disorders and neurotoxicity might be different between females and males (Dluzen et al., 2011; Pena-Bravo et al., 2019; Daiwile et al., 2022). All of the neurospheres used in this study were taken from fetal hippocampi. Because too small to identify their gender, we thus dissociated the mixed hippocampus of both gender. Despite the undetected gender, all samples came from half male and half female according to the Mendel ratio. Hence, the METH-induced cell cycle arrest and aberrant neuronal differentiation may widely mediate the toxicity in both genders.

In addition to the aberrant differentiation of neurons, METH clearly changed the morphology of astrocytes of neurospheres. Astrocytes frequently differ in shape, gene expression, metabolism, and a wide range of other characteristics to adjust their functions. Due to their heterogeneous nature, astrocytes are divided into several subpopulations, including protoplasmic astrocytes located in gray matter (GM) and fibrous astrocytes located in white matter (WM). Protoplasmic astrocytes have rounded somata and highly branched processes, whereas fibrous astrocytes have cell bodies that are more elongated and processes that run parallel to neuronal axons in WM (Grosche et al., 1999; Köhler et al., 2021; Köhler et al., 2023). Similar to the morphology of protoplasmic astrocytes in the brain, the protoplasmic astrocytes in the cultured neurospheres present flat and round, with large cell bodies, wide cytodendrites, and few cytodendritic branches (Liu et al., 2012). In this study, we also visualized that most of the astrocytes in the control neurospheres were flat and round with none or few cytodendritic branches. However, METH exposure caused increased astrocytes with elongated processes. These data suggest that METH might cause a decrease in protoplasmic astrocytes while an increase in fibrous astrocytes in the neurospheres. Because the maintenance of astrocyte morphologies is crucial for proper neural development and maintenance of the brain homeostasis (Matyash and Kettenmann, 2010; Köhler et al., 2021). By providing a physical scaffold for migrating neurons, astrocytes guide neuronal migration during the brain development (Cárdenas et al., 2014). For instance, protoplasmic astrocytes participated in the survival, maturation, and neurogenesis of dopaminergic neurons through secreting neurotrophic factors. Fibrous astrocytes are specialized for maintaining myelin and optimizing nerve signal conduction (Köhler et al., 2021). The changed ratio of astrocytes reflected the abnormal differentiation of astrocytes following METH exposure. Moreover, the decreased number of protoplasmic astrocytes in the METH-treated group also reflected attenuated proliferation upon METH treatment. Thinking above, we consider that in addition to the abnormalities in neurons, the altered morphology of astrocytes may also attribute to the neurodevelopmental toxicity caused by METH.

Besides the cell cycle arrest and aberrant cell differentiation, the accumulated oxidative stress and increased cell apoptosis were also detected in the METH-exposed neurospheres. Consistent with the reported mechanism underlying METH toxicity (Potula et al., 2010; Lenzi et al., 2022), our results also support the engagement of the oxidative stress/mitochondrial mechanism to drive neurodevelopmental impairment. Because both GO and KEGG analysis pointed to the significance of the cell cycle in the METH-caused toxicity in the neurospheres, we proposed that the aberrant cell cycle progression may be primarily affected following the METH exposure, and the abnormal cell differentiation, attenuated migration ability, accumulated ROS, and apoptosis were then initiated.

Overall, we investigated the mechanism underlying the neurodevelopmental toxicity of METH in the neurosphere *in vitro*. The results show for the first time that METH exposure disturbs the cell cycle exit from the self-renewing process and causes aberrant neuronal differentiation, thereby impairing the coordination of neurosphere migration and differentiation during the neurodevelopmental processes. Collectively, our findings shed light on the underlying physiopathological mechanism underlying METH-induced neurodevelopmental deficits.

## Data Availability

The raw sequence data generated during the current study are available in the SRA database (accession number PRJNA999629), https://www.ncbi.nlm.nih.gov/bioproject/PRJNA999629.

## Supplementary material

SUPPLEMENTARY FIGURE S1 METH treatment inhibited Notch signaling in neurospheres. **(A)** The bar graphs display the transcriptional levels of the downstream genes of Notch signaling. n = 4 per group. Ordinary one-way ANOVA, for *Hes1: F_treatment_* (2,9) = 27.35, *P* = 0.0001; *Hes5: F_treatment_* (2,6) = 9.350, *P* = 0.0143; *Hey1: F_treatment_* (2,9) = 21.61, *P* = 0.0004. Compared to the control group, **P* < 0.05, ***P* < 0.01, and ****P* < 0.001. **(B)** Representative images present the classification of astrocytes. Astrocytes were visualized by staining with GFAP and classified into three groups based on their branches: those with no branches, those with long branches below 3, and those with long branches above 3.

## Abbreviations

METH, Methamphetamine; 3D, three-dimensional; GCNs, granule cell neurons; 2D, two-dimensional; CNS, central nervous system; NSCs/NPCs, neural stem and progenitor cells; RT-qPC, R, real-time quantitative-PCR; SDS - PAGE, sodium dodecyl sulfate-polyacrylamide gel electrophoresis; PVDF, polyvinylidene difluoride; DAPI, 4′,6-diamidino-2-phenylindole; ANOVA, one-way analysis of variance; RNA-seq, RNA sequencing; TPM, transcripts per million; PCA, principal component analysis; DEGs, differentially expressed genes; FC, fold change; GO, gene ontology; PI, propidium iodide; PPI, protein-protein interaction; GM, grey matter; WM, white matter; ASD, autism spectrum disorder; AD, Alzheimer’s disease; DHE, dihydroethidium.
