# Supplementary figures and images for: Methamphetamine exposure drives cell cycle exit and aberrant differentiation in rat hippocampal-derived neurospheres
Source: Front Pharmacol. 2023 Sep 19;14:1242109. doi: 10.3389/fphar.2023.1242109 (PMC10546213; doi:10.3389/fphar.2023.1242109)

Figure S1. The original western blot for three repeats

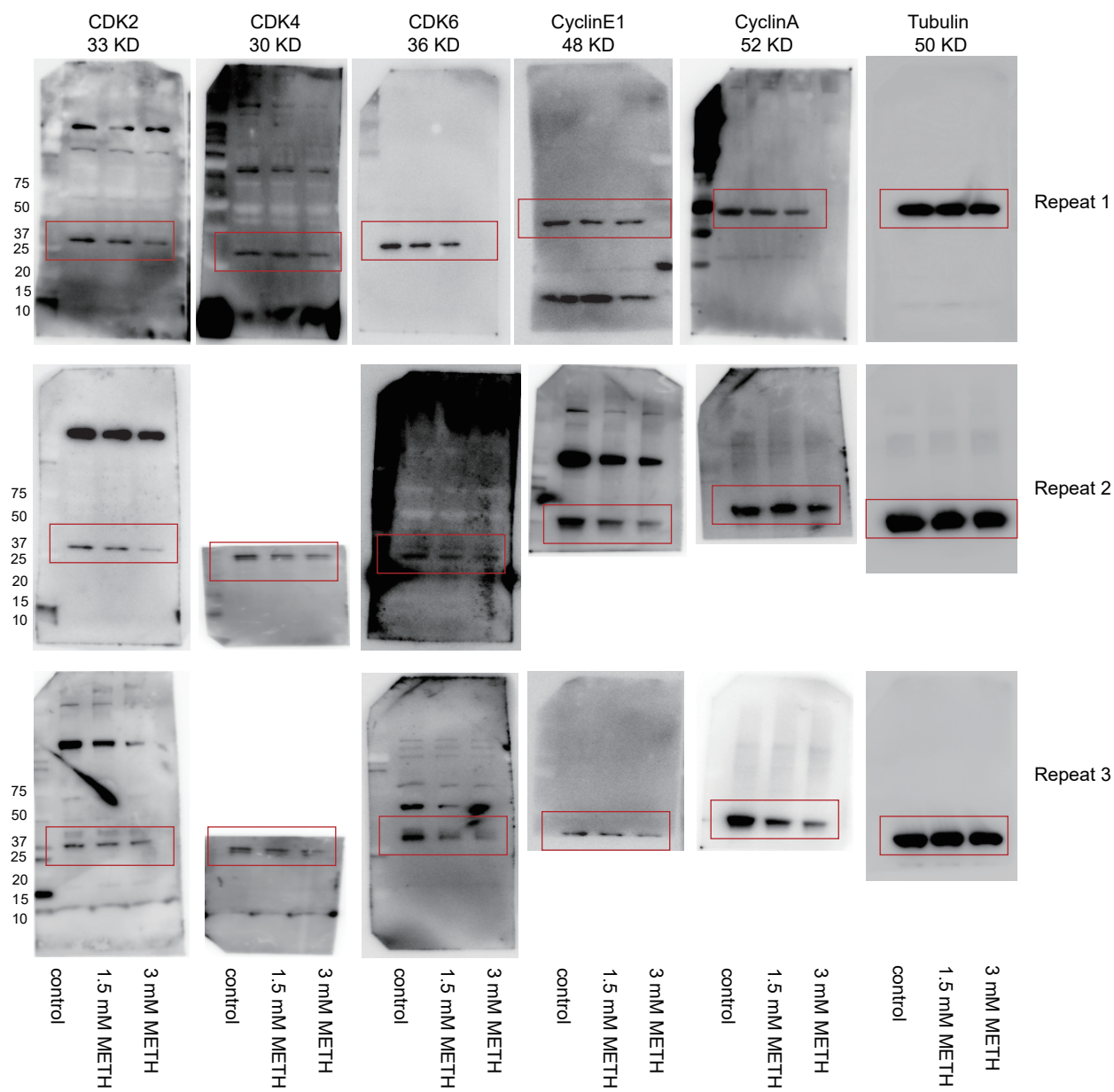

Supplement: Supplementary file 1 [file DataSheet1.PDF]

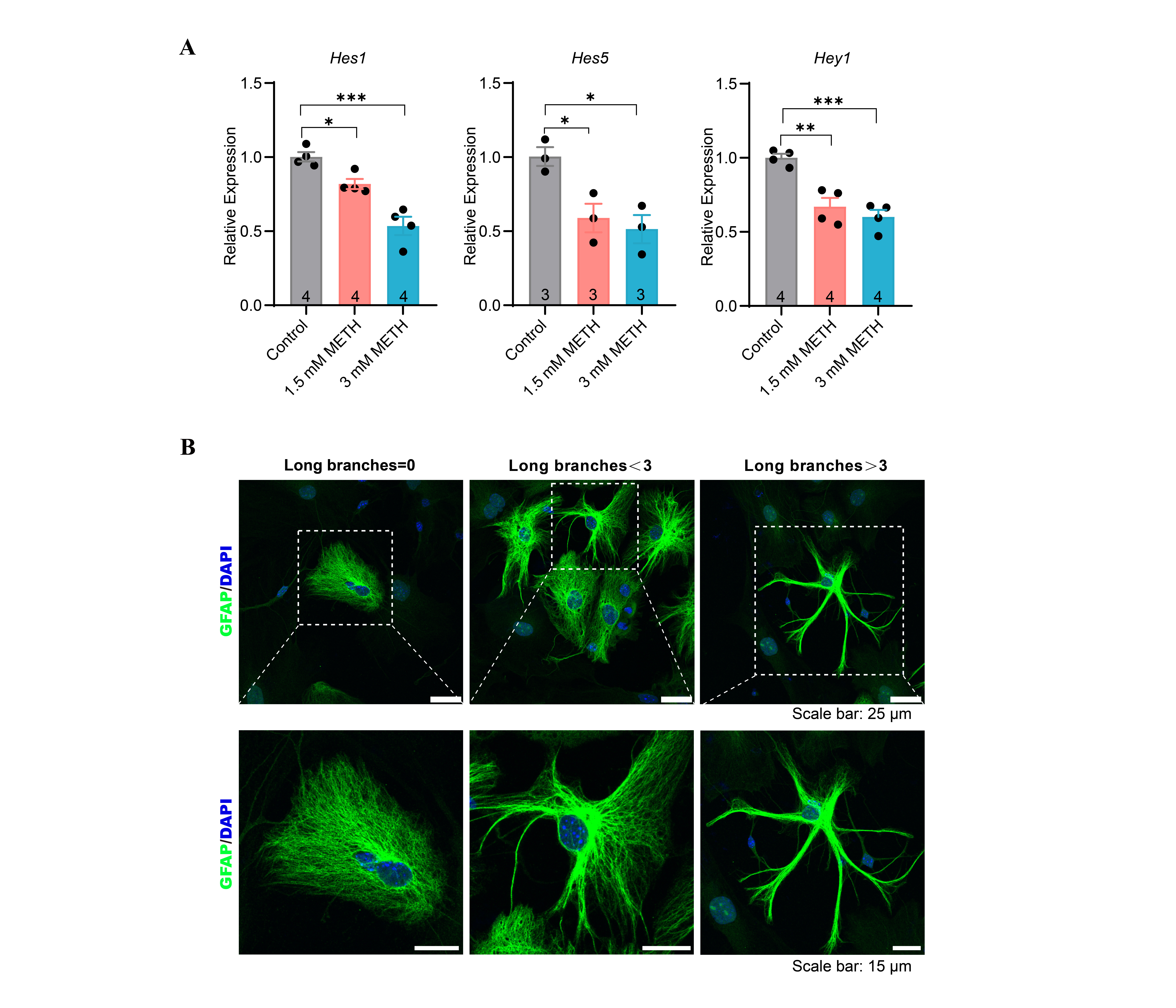

Supplement: Supplementary file 3 [file Image1.jpg]
